# Dimethyl phthalate destroys the cell membrane structural integrity of *Pseudomonas fluorescens*

**DOI:** 10.3389/fmicb.2022.949590

**Published:** 2022-08-22

**Authors:** Wenjing Chen, Ruxin Guo, Zhigang Wang, Weihui Xu, Yunlong Hu

**Affiliations:** ^1^College of Life Sciences, Agriculture and Forestry, Qiqihar University, Qiqihar, China; ^2^Heilongjiang Provincial Technology Innovation Center of Agromicrobial Preparation Industrialization, Qiqihar, China; ^3^Center for Ecological Research, Northeast Forestry University, Harbin, China

**Keywords:** dimethyl phthalate, *Pseudomonas fluorescens*, cell micro-interface, oxidative stress, toxicological effect

## Abstract

A Gram-negative bacteria (*Pseudomonas fluorescens*) was exposed to different concentrations (0, 20, and 40 mg/L) of dimethyl phthalate (DMP) for 8 h, and then Fourier transform infrared spectroscopy (FTIR) analysis, lipopolysaccharide content detection, analysis of fatty acids, calcein release test, proteomics, non-targeted metabolomics, and enzyme activity assays were used to evaluate the toxicological effect of DMP on *P. fluorescens*. The results showed that DMP exposure caused an increase in the unsaturated fatty acid/saturated fatty acid (UFA/SFA) ratio and in the release of lipopolysaccharides (LPSs) from the cell outer membrane (OM) of *P. fluorescens*. Moreover, DMP regulated the abundances of phosphatidyl ethanolamine (PE) and phosphatidyl glycerol (PG) of *P. fluorescens* and induced dye leakage from an artificial membrane. Additionally, excessive reactive oxygen species (ROS), malondialdehyde (MDA), and changes in antioxidant enzymes (i.e., catalase [CAT] and superoxide dismutase [SOD]) activities, as well as the inhibition of Ca^2+^-Mg^2+^-ATPase and Na^+^/K^+^-ATPase activities in *P. fluorescens*, which were induced by the DMP. In summary, DMP could disrupt the lipid asymmetry of the outer membrane, increase the fluidity of the cell membrane, and destroy the integrity of the cell membrane of *P. fluorescens* through lipid peroxidation, oxidative stress, and ion imbalance.

## Introduction

Phthalic acid esters (PAEs) as plasticizers have caused ecological and environmental risks (Chen et al., 2020), of which dimethyl phthalate (DMP) is the most extensively used compound for manufacturing various products (Li et al., 2016; Zhang et al., 2016), and has also been widely detected in the ecological environment, such as surface water, groundwater, atmosphere, and soil (Wang et al., 2015a; Zhang et al., 2015). Based on the published literatures, the acute toxicities of DMP to different aquatic organisms were in the range of 29.0–337.0 mg/L (Gao et al., 2021), and the concentrations of DMP up to 2, 50, and 250 mg/kg in sediment, soil, and sludge, respectively (Pietrini et al., 2022). Additionally, DMP can accumulate in the environment and organisms because of its intractable degradability (Wang et al., 2018; You et al., 2019). Therefore, the toxicological effects of DMP on the ecosystem and organisms were studied.

Microbes can sense and respond to changes in the surrounding environment, in which cell walls and membranes are important barriers against external disturbances, sustain basic cell metabolism and protect cells (Auer and Weibel, 2017), and the effect of DMP on the micro-interface of Gram-positive and Gram-negative bacteria has been reported. For instance, DMP exposure disrupted the cell membrane of *Escherichia coli* K-12, including surface properties and membrane compositions (Wang et al., 2019b); when DMP contacted with *Staphylococcus aureus*, some of it was accumulated on the surface of the cell membrane and destructed the permeability of the cell membrane, and some entered the cell and interacted with macromolecular substances (Zhu et al., 2021). In agricultural systems, *Pseudomonas fluorescens* (*P. fluorescens*) plays a vital role in soil pollution repair and biological fertility (Bensidhoum et al., 2016; Shinde et al., 2017; Lopes et al., 2018), and the abundance of *P. fluorescens* has been significantly reduced by 20 and 40 mg/kg of soil contamination with DMP (Wang et al., 2015a, 2018). Furthermore, 20 and 40 mg/L of DMP caused the morph of the cell membrane, which in turn inhibited the growth and glucose utilization of *P. fluorescens* (Wang et al., 2019a). Thus, the mechanism of DMP on membrane damage of *P. fluorescens* was researched in this study, which provides a theoretical basis for understanding the impact of DMP on beneficial soil bacteria, and provides a public alerting function for understanding the influence of DMP on the soil environment.

We hypothesized that DMP affected the structure of cell walls and membranes of *P. fluorescens*, leading to the inhibition of key enzyme activities of the cell membrane. In this study, we used Fourier transform infrared spectroscopy (FTIR), lipopolysaccharide content detection, analysis of fatty acids, liposome preparation, calcein release test, proteomics, non-targeted metabolomics, and enzyme activity assays to evaluate the impact of DMP on the structure of cell walls and membranes of *P. fluorescens*, the results of which are expected to provide additional data and a theoretical basis for identifying the toxic mechanism of DMP to microorganisms and evaluating their risks to the environment.

## Materials and methods

### Materials

Information on the test organism, *P. fluorescens* ATCC 13525, and biochemical reagents [DMP, acetone, phosphatidylglycerol (PG), cholesterol, cardiolipin (CL), and phosphatidylethanolamine (PE)] is provided in Supplementary Table 1.

Dimethyl phthalate solutions were prepared in acetone at a ratio of 1:9 (v/v), and then the DMP solution was added to Luria–Bertani medium (LB) at final concentrations of 0, 20, and 40 mg/L, with each treatment performed in triplicate. After autoclaving and cooling, the activated *P. fluorescens* were inoculated at a 1% dose. The bacteria were grown at 30°C for 8 h with shaking at 130 rpm.

### Fourier transform infrared spectroscopy (FTIR) analysis

After 8 h of incubation, the P. *fluorescens* was collected by centrifuging (10,000 rpm, 30 min, 4°C), and then washed three times with physiological saline solution (Liu G. et al., 2016). After being freeze-dried under a vacuum, the bacteria were mixed with KBr, and the spectral scanning was within the range of 400–4,000 cm^**–**1^ by FTIR (Spotlight 400, USA).

### Extraction and purification of lipopolysaccharides (LPSs)

Lipopolysaccharides (LPSs) were collected by a modification of the thermal phenol method (Wang et al., 2015b). The wet cells were gathered by centrifuging (6,000 rpm, 10 min). After resuspending in sterile deionized water, an equal volume of 90% aqueous phenol was added, and the mixture was stirred vigorously (70°C, 30 min). The aqueous phase was centrifuged and collected, and an equal volume of deionized water was added and the extraction was repeated. Then, the aqueous phase was collected. After being centrifuged at 8,000 rpm for 15 min, each suspension was mixed with 1 ml of 3 M sodium acetate. Subsequently, LPS was precipitated by adding two volumes of ethanol and lyophilized (Ahamad and Katti, 2016).

### Extraction and analysis of fatty acids

The bacteria were collected, washed two times, and resuspended in sterilized deionized water. After the saponification at 100°C for 25 min with 1 ml of 15% NaOH-methanol solution, 2 ml of 25% HCl-methanol solution was added, and then incubated at 80°C for 10 min. Subsequently, the mixture was mixed with 2 ml of 1:1(v/v) hexane: methyl tert-butyl ether, and the organic phase was concentrated to 0.5 ml under a stream of nitrogen gas. Finally, the fatty acid composition was separated and quantified using a GC-MS 6890-5975 system (Agilent Technologies, Palo Alto, CA, USA) (Wang et al., 2019).

### Liposome preparation and calcein release test

Calcein-loaded liposomes were prepared according to literature methods with minor modifications (Pu and Tang, 2017). After dissolving liposomes in chloroform, the solution was dried by rotary evaporation to a thin film, after which a dye solution (60 mM calcein, 50 mM TES, 100 mM NaCl, and pH 7.4) was added. The mixture was repeated by blow until homogeneous, and then extruded 11 times using a 0.22 mm polycarbonate membrane. Subsequently, DMP stock solutions with various concentrations (0, 20, and 40 mg/L) were added to the purified calcein liposome suspension, and the leakage of calcein was measured by an F-7000 fluorescence spectrophotometer (HITACHI, Japan). The excitation and emission wavelengths were 485 and 515 nm, respectively. To induce 100% dye release, 10 ml of 10% (v/v) Triton X-100 was added to dissolve the vesicles for 18 min. The percentage leakage value was calculated using the following formula:


(1) $$\begin{array}{r} dye\;leakage\;\left(\%\right)\;=\;100\times \left(F-F0\right)/\left(Ft-F0\right) \end{array}$$

where, F is the fluorescence intensity by DMP treatment, Ft is the fluorescence intensity corresponding to 100% leakage, and F0 is the fluorescence intensity of the intact liposome.

### Determination of oxidative stress

The generation of reactive oxygen species (ROS) and malondialdehyde (MDA), and the activities of superoxide dismutase (SOD) and antioxidant enzymes catalase (CAT) were measured using assay kits (Supplementary Table 2) (Chen et al., 2016; Ulloa-Ogaz et al., 2017).

According to the operating instructions of the assay kits (Nanjing Jiancheng Bioengineering Institute, Nanjing, China), the content of MDA and the activities of SOD and CAT were calculated based on Eqs. (2–4) below:


(2) $$\begin{array}{r} MDA\;\left(nmol/mg\;prot\right)=\frac{6.45\times \left(OD_{532\;nm}-OD_{600\;nm}\right)}{P} \end{array}$$

(3) $$\begin{array}{r} SOD\;\left(U/mg\;prot\right)=\frac{\left(OD1-OD2\right)\times 134\times P}{OD1} \end{array}$$

(4) $$\begin{array}{r} CAT\;\left(U/mg\;prot\right)=C\times \frac{250}{T}\times 0.05\times P \end{array}$$

where, P is crude enzymes concentration, which is measured by the Bradford method using bovine serum albumin (BSA) as a standard, OD1 is the value of the control group, OD2 is the value of the sample in this experiment, C is the consumption content of H_2_O_2_, and T is the response time.

### Effect of DMP on the key enzyme activity of *P. fluorescens* cell membrane

The ATPase activity was measured using an ATPase assay kit (Supplementary Table 2) (Waugh, 2019). The activity of ATPase was calculated in OD_660_ nm based on Eqs. 5 and 6:


(5) $$\begin{array}{l} Na^{+}-K^{+}-ATPase\left(U/mgprot\right)\;=\;\frac{OD3\;-\;OD2}{OD1}\times \\ \;\left[46.8÷P\right] \end{array}$$

(6) $$\begin{array}{l} Ca^{2+}-Mg^{2+}-ATPase\left(U/mgprot\right)\;=\;\frac{OD4-OD2}{OD1}\times \\ \;\left[46.8÷P\right] \end{array}$$

Where, OD1 is the standard reference, OD2 is the blank value, OD3 and OD4 are the measured values, and P is the crude enzyme concentration.

### Multiomics analysis and statistical analyses

After 8 h of incubation, the *P. fluorescens* were collected and then washed three times with sterile water for proteomics analysis. Three replicates were used for each treatment and all samples were treated with an appropriate buffer which contained a cocktail (1% SDS, 200 mM DTT, 50 mM Tris-HCl, and pH 8.8) at 100°C for 10 min, then transferred to the ice for 30 min. After each suspension was centrifuged and collected, a 5-fold volume of pre-cooled acetone was added to precipitate protein at −20°C overnight. Subsequently, the mixture was centrifuged, and the precipitate was collected and washed two times with 90% acetone. The precipitate was then resuspended in lysis buffer (1% SDS, 8 M urea, cocktail) and then sonicated for 3 min. Furthermore, the concentration of protein in all samples was determined using the bicinchoninic acid (BCA) method, and the protein was digested with a trypsin solution. The digested peptides were separated and analyzed using a nanoLC-MS/MS (Thermo Fisher Scientific, Massachusetts, USA). Finally, the tandem mass spectrometry (MS/MS) spectra were searched by ProteomeDiscovererTM software against *Pseudomonas fluorescens* database. The highest score for a given peptide mass was used to identify the parent protein (Thippakorn et al., 2018).

The metabolites in 50 mg samples (lyophilized supernatant) were extracted using 1 ml of methanol: water (4:1, v/v) solution. In total, five replicates were used for each treatment, and all samples were ground at 60 Hz for 1 h. Before centrifugation at 12,000 rpm for 15 min at 4°C, the supernatant was pipetted into a 5-mm vial for subsequent liquid chromatography–mass spectrometry (LC-MS) analysis. Briefly, a chromatographic column (100 mm × 2.1 mm, i.d., 1.7 μm; Waters, Milford, USA) separation was performed with acetonitrile and 0.1% formic acid as the mobile phase. A 5–95% gradient of acetonitrile over 16 min was used, with an injection volume of 20 μl and a flow rate of 0.4 ml/min. The positive ion mode and negative ion mode were performed by mass spectrometry (MS) using a scan time of 0.03 s and a 50–1,000 m/z scan range. In addition, Mass Profiler software (Agilent, California, USA) was used to extract features from the metabolomics data, and the differential metabolites were identified based on the combination of the variable importance in projection (VIP) value obtained from the PLS-DA model and a *p*-value determined based on the raw data (Thippakorn et al., 2018).

Moreover, one-way ANOVA followed by Tukey's HSD test was performed to detect the significant differences among all the experimental treatments, with *p* < 0.05 denoting significance, and all analyses were performed in R software.

## Results

### Effect of DMP on the cell wall of *P. fluorescens*

**Figure 1:**
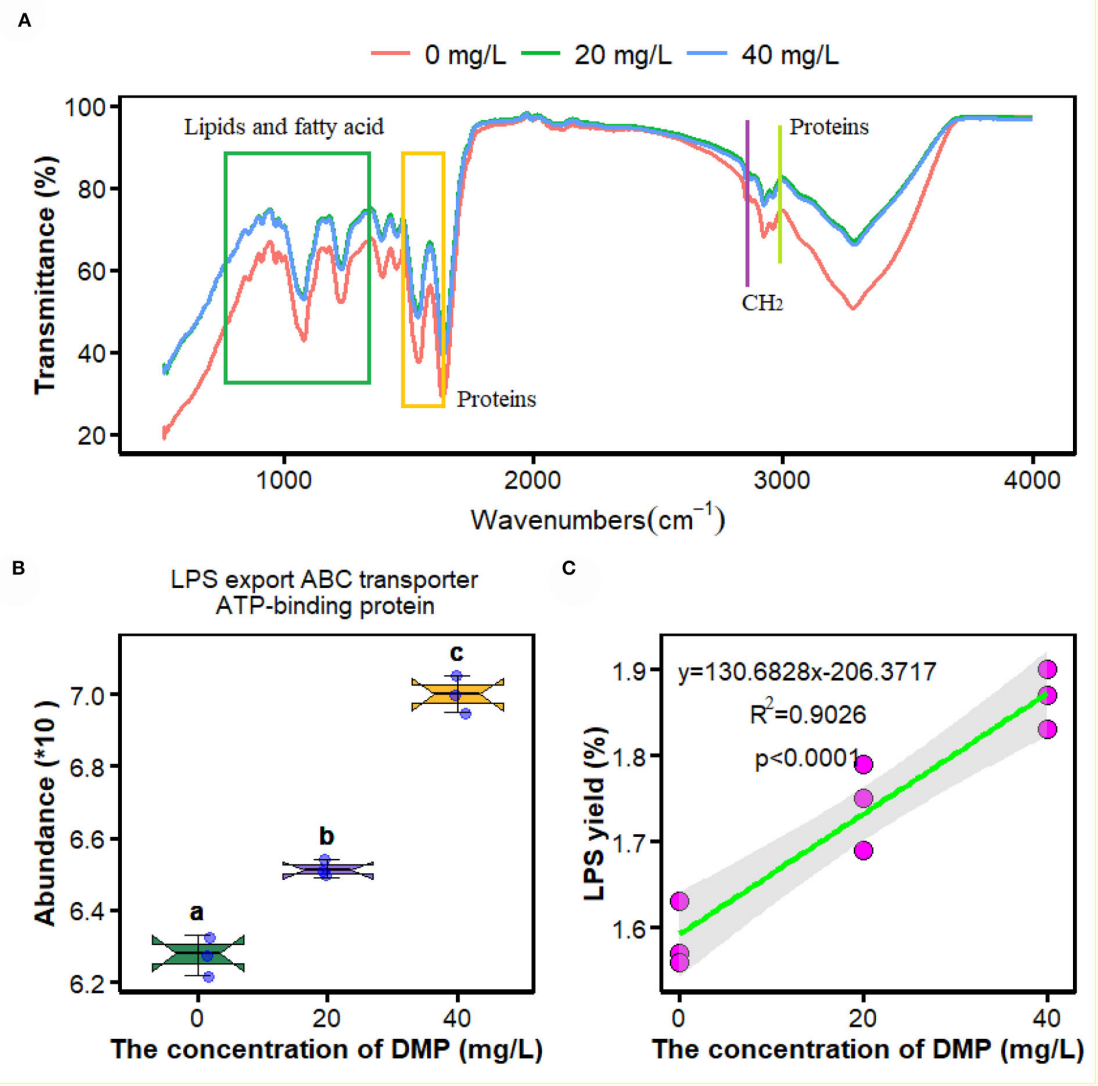
Effects of dimethyl phthalate (DMP) on the cell surface functional groups, lipopolysaccharide (LPS) transporter and LPS content of *Pseudomonas fluorescens (P. fluorescens)*. **(A)** shows DMP changed the cell surface functional groups; **(B)** shows that DMP increased the expression of LPS export ATP-binding cassette (ABC) transporter ATP-binding protein abundance through proteomics, and **(C)** shows LPS content of *P. fluorescens*. Different letters represent significant differences between treatments.

Fourier transform infrared spectroscopy is an efficient tool to examine DMP-induced toxicity in *P. fluorescens*. In this study, compared with the control samples (0 mg/L), spectral differences were observed mainly in the absorption bands of lipids and fatty acids in the range of 1,500–1,640 cm^**–**1^ and 3,014 cm^**–**1^, and those proteins in the range of 750–1,358 cm^**–**1^. However, the FTIR spectroscopy of *P. fluorescens* between 20 and 40 mg/L DMP treatment was no different in Figure 1A.

To evaluate how DMP influences the micro-interface of *P. fluorescens*, the proteomics and metabolomics were measured. One advantage of this method was that all the omics analyses used the same samples, which could provide strong links between the datasets. As depicted below and visible from the principal component analysis (PCA; Supplementary Figures 1A,B), DMP profoundly influenced the proteome and metabolome of *P. fluorescens*, which indicated that the protein expression and functional characteristics of *P. fluorescens* were significantly altered by DMP treatment. Moreover, the proteomics results of all significantly changed proteins are presented in Supplementary Table 3.

The proteomics analysis and the yield of crude LPS were used to investigate the effects of DMP treatment on the LPS transporter and the yield of *P. fluorescens*. In Figure 1B, the abundance of LPS export ATP-binding cassette (ABC) transporter ATP-binding protein was increased by DMP treatment with 20 and 40 mg/L. At the same time, the content of crude LPS was enhanced, and the increasing concentration of DMP was positively correlated with the yield of crude LPS in Figure 1C (*R*^2^ = 0.9026, *p* < 0.0001).

### Effect of DMP on fatty acid in the cell membrane

**Figure 2:**
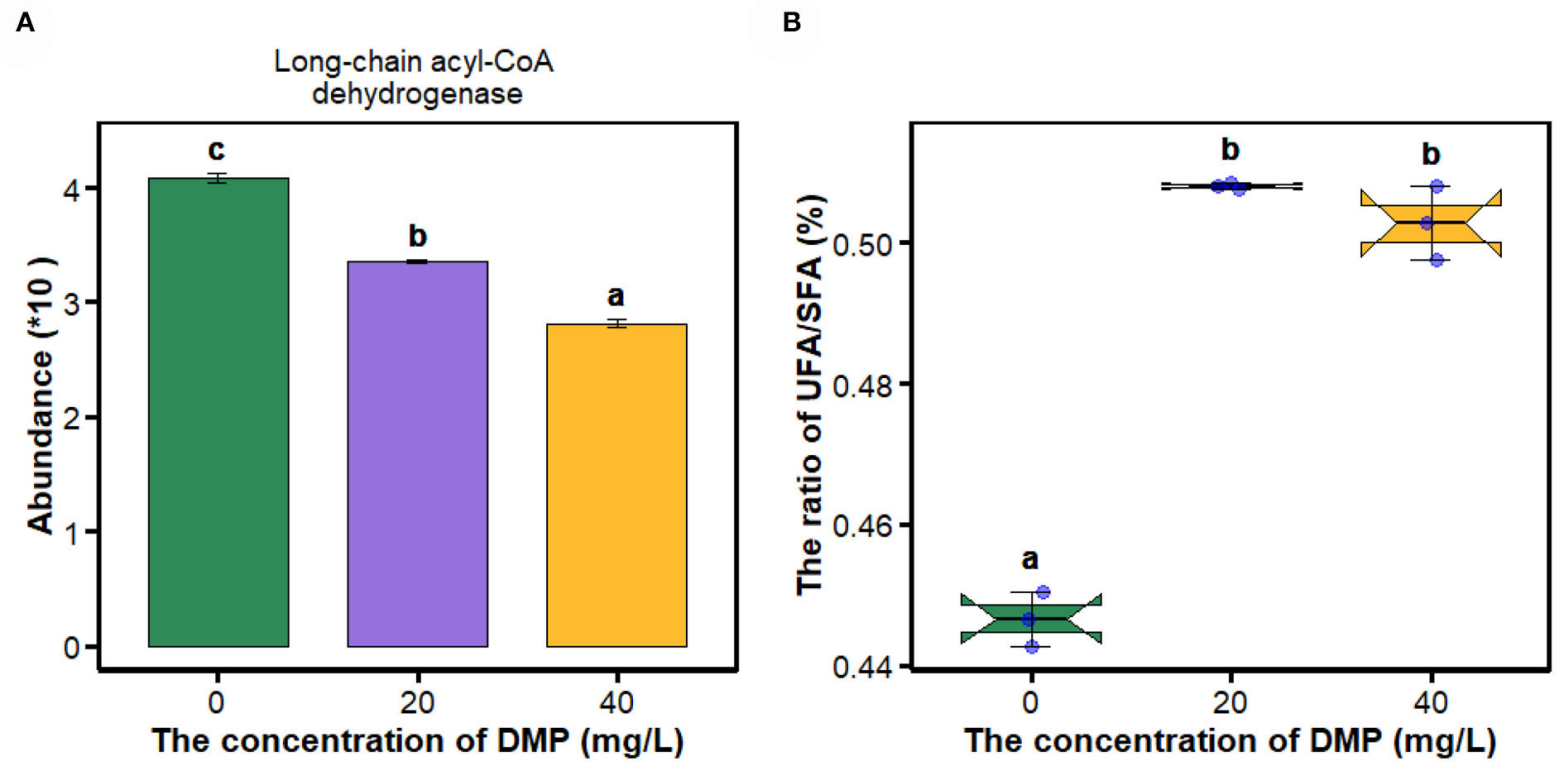
The expression of proteins in the fatty acid metabolism pathway and the ratio of UFA/SFA of *P. fluorescens* were altered by DMP contamination. **(A)** shows that DMP inhibited the expression of long-chain acyl-CoA dehydrogenase abundance through proteomics, and **(B)** shows that the ratio of UFA/SFA of *P. fluorescens* was changed by DMP treatment. Different letters represent significant differences between treatments.

Fatty acids as energy substrates are metabolized and synthesized during biological responses. In Figure 2A, the abundance of long-chain acyl-CoA dehydrogenase was decreased in *P. fluorescens* with the increase in DMP concentration. Then, the ratio of UFA/SFA was analyzed as shown in Figure 2B. The ratio of UFA/SFA of *P. fluorescens* was increased by DMP treatment with 20 and 40 mg/L, and these **two** DMP treatments were not different.

### Effect of DMP on the phospholipid and integrity of cell membrane of *P. fluorescens*

**Figure 3:**
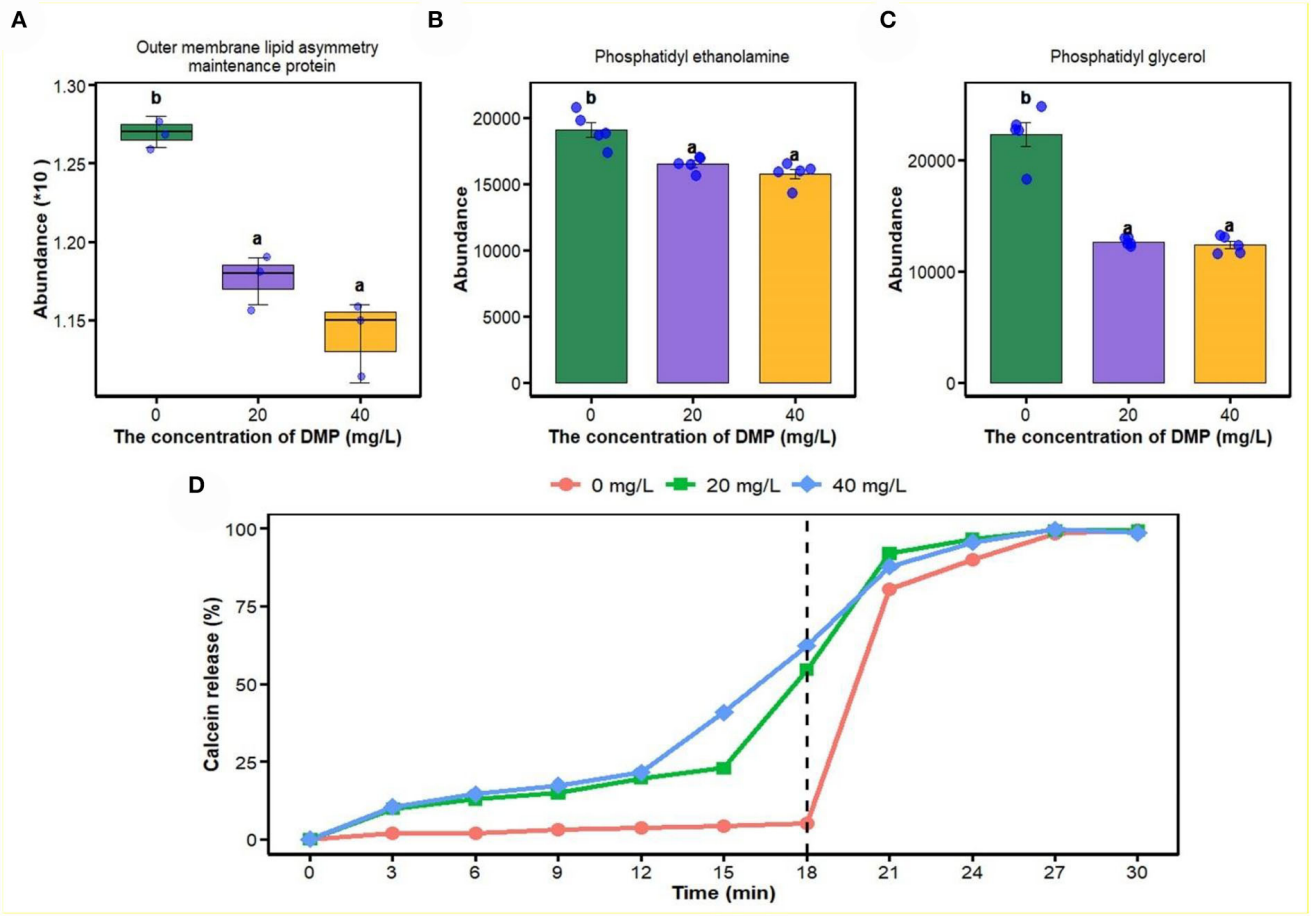
The effect of DMP on the phospholipid and integrity of cell membrane of the *P. fluorescens*. **(A)** indicates that DMP inhibited the expression of outer membrane (OM) lipid asymmetry maintenance protein abundance through proteomics; **(B**,**C)** indicate that the abundance of phosphatidyl ethanolamine (PE) and phosphatidyl glycerol (PG) were decreased by DMP treatment through metabolomics; **(D)** indicates that the integrity of the cell membrane of *P. fluorescens* was changed by DMP treatment by the calcein release test. Different letters represent significant differences between treatments.

In this study, proteomics, metabolomics, and artificial plasma membrane were used to study the effect of DMP on the cell membrane, and the result is shown in Figure 3. Compared with 0 mg/L DMP treatment, the expression of outer membrane (OM) lipid asymmetry maintenance protein (Figure 3A), phosphatidyl ethanolamine (PE, Figure 3B), and phosphatidyl glycerol (PG, Figure 3C) were decreased by DMP treatment with 20 and 40 mg/L. Finally, the calcein release test was introduced to study the effect of DMP on the cell membrane structure of *P. fluorescens*, which showed that before the reaction of 18 min, the leakage of calcein in liposomes was 5.2, 54.6, and 62.3%, respectively. However, when 10% Triton X-100 was added, calcein quickly leaked from the liposome and the leakage was completed in 30 min as shown in Figure 3D.

### Effect of DMP on the oxidative reactive kinase system

**Figure 4:**
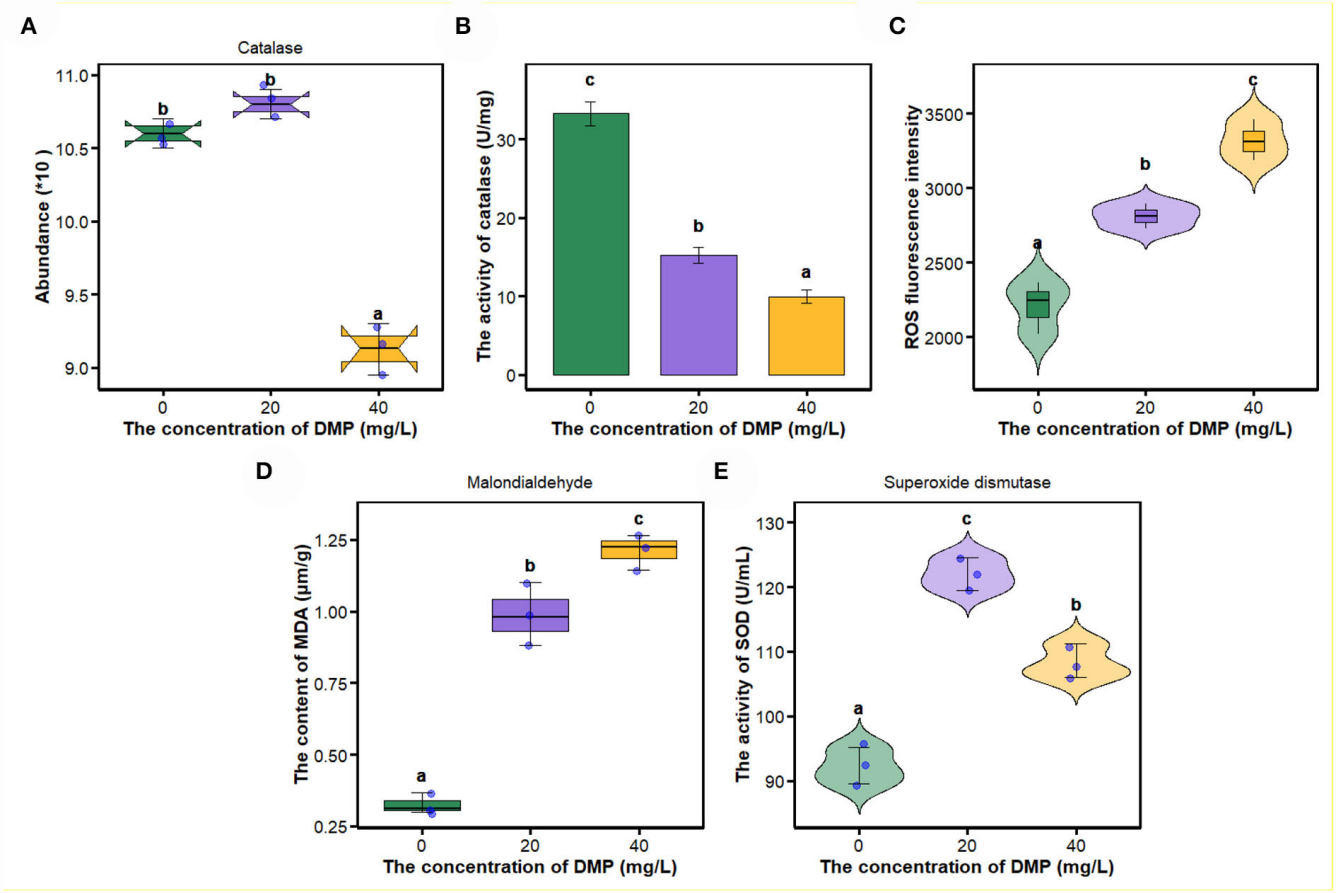
The effect of DMP on the oxidative-reactive kinase system of the *P. fluorescens*. **(A)** indicates that DMP inhibited the expression of catalase abundance through proteomics; **(B,E)** show the activities of catalase (CAT) **(B)** and superoxide dismutase (SOD) **(E)** of *P. fluorescens* was changed by DMP treatment; **(C,D)** show reactive oxygen species (ROS) and malondialdehyde (MDA). Different letters represent significant differences between treatments.

In this study, compared with 0 and 20 mg/L, the abundance and activity of catalase (Figures 4A,**B**) were decreased by DMP treatment with 40 mg/L, and then, the ROS fluorescence intensity, malondialdehyde content, and the activity of superoxide dismutase (SOD) were analyzed, and the results are shown in Figures 4C–E. For ROS fluorescence intensity (Figure 4C) and malondialdehyde content (Figure 4D), conform to dose effects that were increased with the increase of DMP concentration, and the activity of SOD was increased by DMP treatment at 20 and 40 mg/L, with the 20 mg/L being higher than the 40 mg/L.

### Effects of DMP on key enzymes on the cell membrane

**Figure 5:**
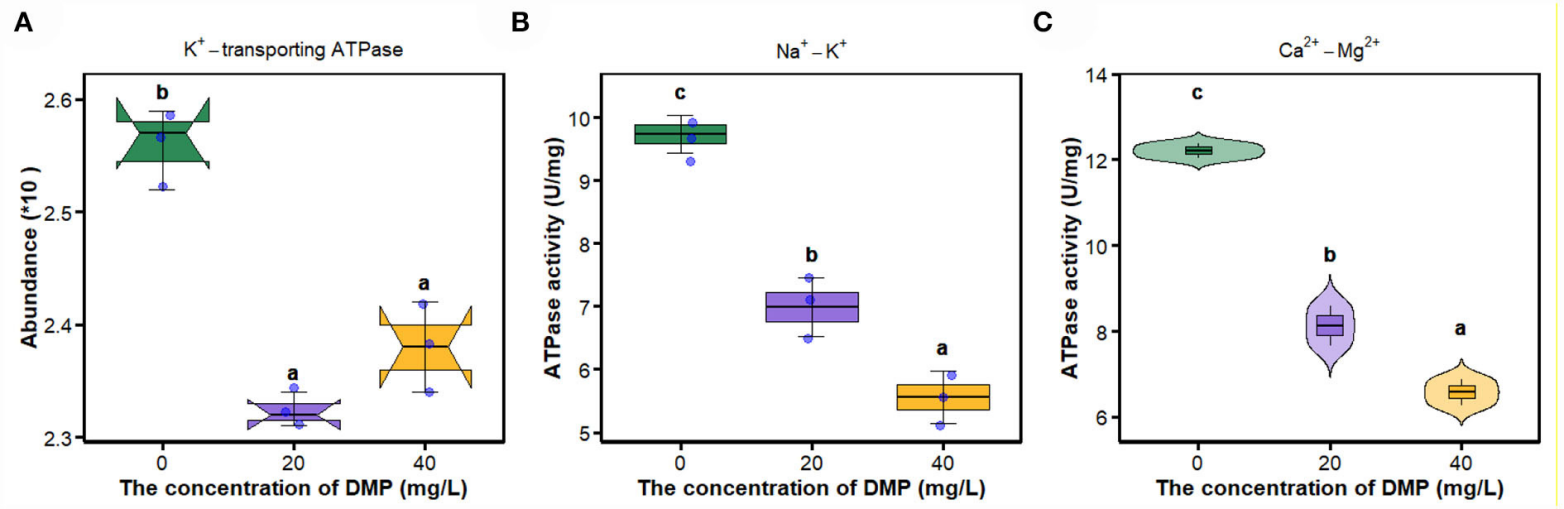
The effect of DMP on key enzymes on the cell membrane of the *P. fluorescens*. **(A)** indicates that DMP inhibited the expression of K^+^-transporting ATPase abundance through proteomics; **(B,C)** indicate that the activities of Na^+^-K^+^-ATPase and Ca^2+^-Mg^2+^-ATPase of *P. fluorescens* were changed by DMP treatment. Different letters represent significant differences between treatments.

In Figure 5A, compared with 0 mg/L DMP treatment, the abundance of K^+^-transporting ATPase was decreased by DMP treatment with 20 and 40 mg/L, and these **two** treatments had no difference, after which the activities of Na^+^-K^+^-ATPase, and Ca^2+^-Mg^2+^-ATPase were studied, and the results are shown in Figures 5B,C. Those activities conform to dose effects that were decreased with the increase of DMP concentration.

## Discussion

Knowledge of the effects of DMP on the micro-interface of microbes has accumulated rapidly over the past few years (Wang et al., 2019a,b; Zhu et al., 2021). In a previous study, DMP induced the deformations of the cell membrane of *P. fluorescens* (Wang et al., 2019a). Therefore, in this study, we focused on the mechanism of DMP damage to cell walls and cell membranes and its adverse effect on *P. fluorescens*. FTIR peaks are related to the vibration of a particular chemical bond, and are used to characterize the changes in the macromolecular functional groups on the cell surface (Movasaghi et al., 2008). In Figure 1A, DMP treatment can promote the stretching vibration of protein lipids, and fatty acids of *P. fluorescens*, which indicates that DMP exposure will change the contents of protein, lipids, and fatty acids (Simsek Ozek et al., 2014). Therefore, proteomics, metabolomics, LPS, and fatty acid content were used to study the effect of DMP on the cell micro-interface.

The outer membrane of Gram-negative bacteria provides the cell with a permeability barrier that is effective against external harmful agents, and a tightly packed layer of LPS exists on the surface of the outer membrane plays a key role in this process (Zhao et al., 2011; Cui et al., 2019; Sperandeo et al., 2019; Zhou et al., 2021). After biosynthesis, bacterial LPS are transiently anchored to the outer leaflet of the inner membrane, and the ABC transporter transports LPS molecules to the outer membrane (Luo et al., 2017). In this study, the abundance of LPS export ABC transporter ATP-binding protein (Figure 1B) and the release of LPS from the cell outer membrane of *P. fluorescens* (Figure 1C) was increased by DMP. LPS has high chemical stability after the death of bacterial cells (Pourmadadi et al., 2019), and the number of dead cells was increased by DMP (Wang et al., 2019a), which may be another reason for the increase in LPS.

The cell membrane maintains a dynamic balance and plays a critical role in maintaining a normal state for growth, metabolism, and stress resistance (Li et al., 2019). Many studies have reported the correlation between the cell membrane and active cell responses to environmental stresses. When *Lactobacillus delbrueckii* subspecies bulgaricus CFL1 was exposed to stress conditions, the composition of the lipid bilayer of the cell membrane would maintain an optimal level of fluidity (Gautier et al., 2013). For the *Lactobacillus casei* strains, the acid-resistant mutant strain has a higher proportion of UFA, and better fluidity and integrity than those of the wild type (Wu et al., 2012). In Figure 2B, the ratio of UFA/SFA of *P. fluorescens* was increased by DMP treatment with 20 and 40 mg/L, which is commonly used for assessing the membrane fluidity. A high UFA/SFA ratio is usually associated with high membrane fluidity (Gautier et al., 2013). In Figure 1A, the position of the lipid symmetric CH_2_ stretching vibration band at approximately 2,850 cm^−1^ is a measure of membrane lipid state generally related to membrane fluidity (Gautier et al., 2013). Thus, the fluidity of the cell membrane of *P. fluorescens* was increased by DMP treatment.

The outer membrane (OM) of Gram-negative bacteria is an extremely asymmetric bilayer, which is maintained by the OM phospholipase A2, the LPS palmitoyl transferase, and the maintenance of the outer membrane lipid asymmetry system (Abellón-Ruiz et al., 2017; Yeow et al., 2018). The accumulation of LPS (Figure 1C) and downregulated expression of outer membrane lipid asymmetry maintenance protein (Figure 3A) by DMP treatment, indicated that DMP could disrupt the lipid asymmetry of the outer membrane. In a previous study, *E. coli* balances the quantity of PG and *Pseudomonas aeruginosa* increases the length of the lipid chains as a response to environmental signals (Hoyo et al., 2019). In Figure 3B and C, *P. fluorescens* regulated the abundances of PG and PE as a response to DMP contamination. Moreover, DMP contamination increased the rates of dead cells and hypoactive cells, which indicated that DMP increased the membrane permeability and damaged membrane integrity (Wang et al., 2019a). Furthermore, DMP-induced dye leakage from artificially simulated the liposomes of *P. fluorescens* in Figure 3D, which indicated that the integrity of the cell membrane was compromised by DMP (Sun et al., 2015).

Since lipids are responsible for maintaining the integrity of cell membranes, significant lipid peroxidation changes the assemblage, composition, structure, and dynamics of lipid membranes (Gaschler and Stockwell, 2017). As an end product of lipid peroxidation, the formation of MDA as an index to measure lipid peroxidation (Dubovskiy et al., 2008; Liu N. et al., 2016), and the increased concentration of MDA by DMP treatment in Figure 4D, indicated the DMP pollution resulted in lipid peroxidation of *P. fluorescens*. The aldehyde groups of MDA can interact with amino groups in proteins and phospholipids, which result in altered membrane permeability, lipid organization, and cellular dysfunction (Chauhan et al., 2002). Additionally, high concentrations of ROS cause membrane damage through lipid peroxidation in Figure 4C, resulting in the leakage of cell contents, loss of respiratory activity, and cell death (Yu et al., 2019).

As an important components of the antioxidant defense system in cells, SOD can eliminate excess ROS to maintain intracellular oxidation-reduction equilibrium, and catalyze the dismutation of superoxide into molecular oxygen and hydrogen peroxide (H_2_O_2_) (Arts et al., 2015; Liu N. et al., 2016; Wu et al., 2017; Wang et al., 2020; Gao et al., 2021). SOD enzyme activity increases after DMP contamination (Figure 4E), which suggested that DMP caused oxidative stress and stimulated SOD activity to scavenge the fast-producing ROS (Wu et al., 2017; Gao et al., 2021). Thus, we assumed that DMP's increased SOD activity would result in an increased H_2_O_2_ concentration. Furthermore, fatty acid oxidation-driven H_2_O_2_ is a major source of oxidative stress, the long-chain acyl-CoA dehydrogenase, which catalyzes a key step in mitochondrial fatty acid oxidation, the expression of which (Figure 2A) is significantly related to H_2_O_2_ (Zhang et al., 2019). In this study, the abundance (Figure 4A) and activity (Figure 4B) of CAT were significantly decreased, as a result of the high level of superoxide radical generation during oxidative stress (Dubovskiy et al., 2008). Excessive ROS inhibits the activities of catalase, resulting in lipid oxidation and the destruction of algae cell membranes (Huang et al., 2021). In sum, lipid oxidation and oxidative stress could be the main mechanisms of DMP-induced cell membrane damage.

The movement of ions through the cell membrane facilitates the regulation of the cell volume, preventing the cell from potentially breaking or collapsing under changing conditions (Pinsky, 2021). The imbalance between the internal and external environment of the cell directly leads to the metabolism of the cell, and the proliferation of the cell is out of control (Xin et al., 2020). Na^+^-K^+^-ATPase transports Na^+^ ions and K^+^ ions against its concentration gradient across the membrane, and preserves the membrane potential and osmotic equilibrium of the cell (Xie and Cai, 2003). Active Ca^2+^ transport across the membrane is carried out by Ca^2+^-Mg^2+^-ATPase (Li et al., 2006). Ca^2+^-Mg^2+^-ATPase and Na^+^/K^+^-ATPase catalyze the hydrolysis of ATP that is coupled to the active transport of Ca^2+^/Mg^2+^and Na^+^/K^+^ across the cell membrane (Chauhan et al., 2002). Studies have shown that the enzyme activity of *E. coli* Na^+^-K^+^-ATPase is inhibited under hexabromocyclododecane (HBCD) stress, and HBCD can combine with Ca^2+^-ATPase, resulting in a decrease in intracellular ATP levels and thus affecting glucose transport (Yang et al., 2020). This study found that DMP significantly inhibits the expression of K^+^-transporting ATPase (Figure 5A) and the activities of Na^+^-K^+^-ATPase (Figure 5B) and Ca^2+^-Mg^2+^-ATPase (Figure 5C) of *P. fluorescens*. The inhibition of these enzymes leads to the instability of ion entry into the cell, then the cell may swell and eventually cause the cell membrane to rupture (Zhou et al., 2011; Xin et al., 2020).

## Conclusion

This study showed that DMP could disrupt the lipid asymmetry of the outer membrane, increase the fluidity of the cell membrane, and destroy the integrity of the cell membrane of *P. fluorescens*; excessive ROS, MDA, and changes in antioxidant enzymes (i.e., CAT and SOD) activities in *P. fluorescens*, which caused lipid peroxidation and oxidative stress, were induced by the DMP; and inhibition of Ca^2+^-Mg^2+^-ATPase and Na^+^/K^+^-ATPase activities in *P. fluorescens*, which reduced ions imbalance by the DMP. Therefore, DMP could destroy the cell membrane structural integrity of *P. fluorescens* through lipid peroxidation, oxidative stress, and ion imbalance.

## Data availability statement

The data presented in the study are deposited in the iProX repository, accession number IPX0003930004. All data was released.

## Author contributions

ZW made substantial contributions to the design, the acquisition, analysis, and interpretation of data for the work. WC and RG performed the experiment and drafted the work. WX and YH revised it critically for important intellectual content. All authors contributed to the article and approved the submitted version.

## Funding

This work was supported by the Natural Science Foundation of China (Grant Nos. 31870493) and the Graduate Innovation Project of Qiqihar University (YJSCX2019047).

## Conflict of interest

The authors declare that the research was conducted in the absence of any commercial or financial relationships that could be construed as a potential conflict of interest.

## Publisher's note

All claims expressed in this article are solely those of the authors and do not necessarily represent those of their affiliated organizations, or those of the publisher, the editors and the reviewers. Any product that may be evaluated in this article, or claim that may be made by its manufacturer, is not guaranteed or endorsed by the publisher.
