# Supplementary figures and images for: Dimethyl phthalate destroys the cell membrane structural integrity of Pseudomonas fluorescens
Source: Front Microbiol. 2022 Aug 22;13:949590. doi: 10.3389/fmicb.2022.949590 (PMC9441906; doi:10.3389/fmicb.2022.949590)

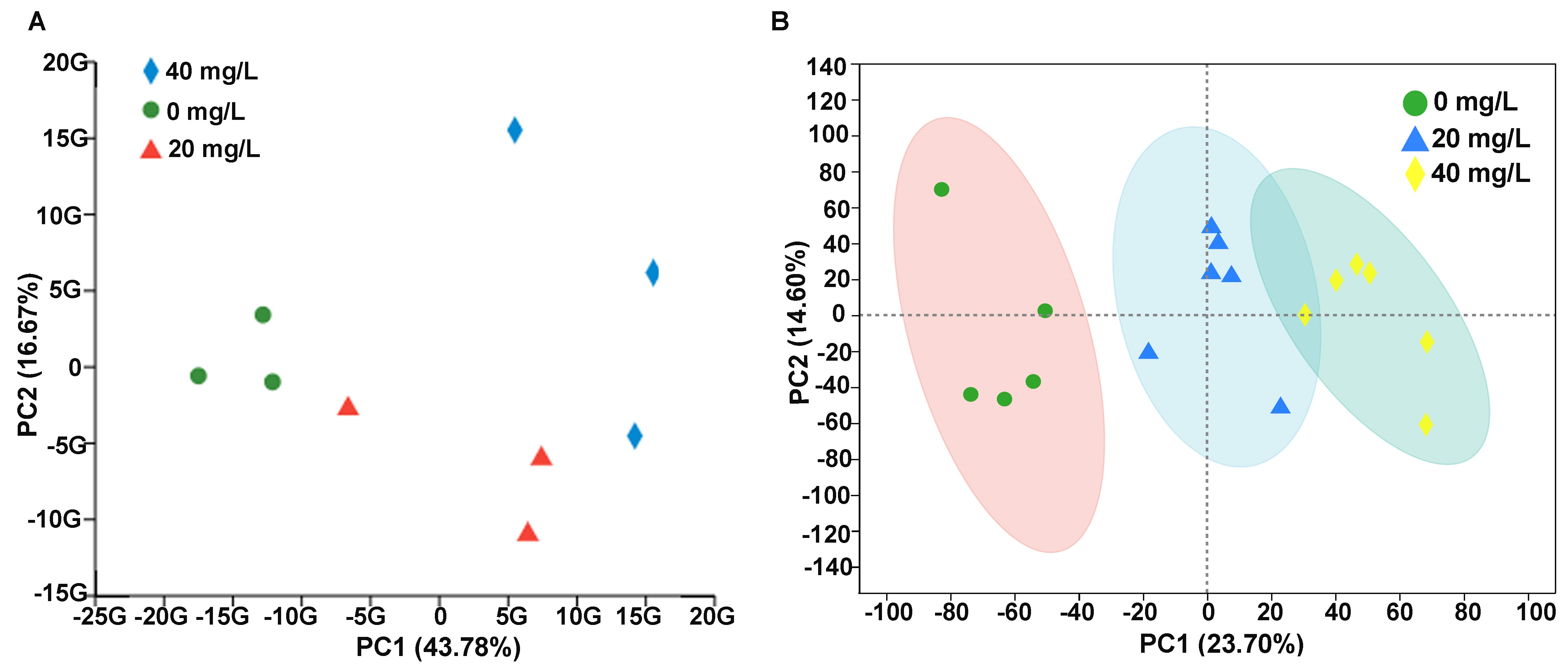

Supplement: Supplementary file 1 [file Image_1.jpg]
