# Supplementary material for: Dimethyl phthalate destroys the cell membrane structural integrity of Pseudomonas fluorescens
Source: Front Microbiol. 2022 Aug 22;13:949590. doi: 10.3389/fmicb.2022.949590 (PMC9441906; doi:10.3389/fmicb.2022.949590)
Supplement: Supplementary file 2 [file Table_1.docx]

Supplementary Material

**Supplementary Table 1.** Information on the test organism and biochemical reagents.

| Products | Purity | Manufacturer |
| --- | --- | --- |
| *P. fluorescens* ATCC 13525 |  | China General Microbiological Culture Collection Center, Beijing, China |
| DMP | > 99.5% | Guangfu Fine Chemical Research Institute, Tianjin, China |
| Acetone | > 99.5% | Traditional Chinese Medicine, Beijing, China |
| PG | > 99.5% | Sigma-Aldrich, Shanghai, China |
| PE | > 99.5% | Sigma-Aldrich, Shanghai, China |
| CL, | > 99.5% | Sigma-Aldrich, Shanghai, China |
