# Supplementary material for: Dimethyl phthalate destroys the cell membrane structural integrity of Pseudomonas fluorescens
Source: Front Microbiol. 2022 Aug 22;13:949590. doi: 10.3389/fmicb.2022.949590 (PMC9441906; doi:10.3389/fmicb.2022.949590)
Supplement: Supplementary file 3 [file Table_2.docx]

Supplementary Material

**Supplementary Table 2.** The information of kit used in measuring the intermediates and enzymes.

| Parameters | Products | Number | Manufacturer | Method |
| --- | --- | --- | --- | --- |
| ROS | ROS assay kit | EOO4-1 | Nanjing Jiancheng Bioengineering Institute, Nanjing, China | The fluorescence probe 2′,7′-dichlorofluorescin-diacetate (DCFH-DA) was used to quantify the generation of ROS, and the excitation wavelength and emission wavelength were 485 nm of 535 nm, respectively (Ulloa-Ogaz et al., 2017). |
| MDA | MDA assay kit | A003-1 | Nanjing Jiancheng Bioengineering Institute, Nanjing, China | MDA was monitored by quantifying thiobarbituric acid (TBA)-reactive substances (Chen et al., 2016) |
| SOD | SOD assay kit | A001-1 | Nanjing Jiancheng Bioengineering Institute, Nanjing, China | SOD activity was assayed using the hydroxylamine reduction method, and the reduction of hydroxylamine by O_2_, which was monitored at 550 nm (Chen et al., 2016) |
| CAT | CAT assay kit | A007-1 | Nanjing Jiancheng Bioengineering Institute, Nanjing, China | CAT activity was measured by monitoring the reduction of H_2_O_2_ in absorbance at 240 nm (Chen et al., 2016) |
| ATPase | ATPase assay kit | A016-1 | Nanjing Jiancheng Bioengineering Institute, Nanjing, China | The activities of Na^+^-K^+^-ATPase and Ca^2+^-Mg^2+^-ATPase were expressed as the releasing amount of inorganic phosphorus, which were monitored at 660 nm (Waugh, 2019) |
